# Junctional adhesion molecule-A deletion increases phagocytosis and improves survival in a murine model of sepsis

**DOI:** 10.1172/jci.insight.156255

**Published:** 2022-08-22

**Authors:** Nathan J. Klingensmith, Katherine T. Fay, David A. Swift, Julia M.R. Bazzano, John D. Lyons, Ching-wen Chen, Mei Meng, Kimberly M. Ramonell, Zhe Liang, Eileen M. Burd, Charles A. Parkos, Mandy L. Ford, Craig M. Coopersmith

**Affiliations:** 1 Department of Surgery and Emory Critical Care Center, Emory University School of Medicine, Atlanta, Georgia, USA.; 2 Department of Critical Care Medicine, Ruijin Hospital, Shanghai Jiao Tong University School of Medicine, Shanghai, China.; 3 Department of Pathology and Laboratory Medicine, Emory University School of Medicine, Atlanta, Georgia, USA.; 4 Department of Pathology, University of Michigan, Ann Arbor, Michigan, USA.; 5 Department of Surgery and Emory Transplant Center, Emory University School of Medicine, Atlanta, Georgia, USA.

**Keywords:** Infectious disease, Bacterial infections, Innate immunity, Tight junctions

## Abstract

Expression of the tight junction–associated protein junctional adhesion molecule-A (JAM-A) is increased in sepsis, although the significance of this is unknown. Here, we show that septic JAM-A ^–/–^ mice have increased gut permeability, yet paradoxically have decreased bacteremia and systemic TNF and IL-1β expression. Survival is improved in JAM-A^–/–^ mice. However, intestine-specific JAM-A^–/–^ deletion does not alter mortality, suggesting that the mortality benefit conferred in mice lacking JAM-A is independent of the intestine. Septic JAM-A^–/–^ mice have increased numbers of splenic CD44^hi^CD4^+^ T cells, decreased frequency of TNF^+^CD4^+^ cells, and elevated frequency of IL-2^+^CD4^+^ cells. Septic JAM-A^–/–^ mice have increased numbers of B cells in mesenteric lymph nodes with elevated serum IgA and intraepithelial lymphocyte IgA production. JAM-A^–/–^ × RAG^–/–^ mice have improved survival compared with RAG^–/–^ mice and identical mortality as WT mice. Gut neutrophil infiltration and neutrophil phagocytosis are increased in JAM-A^–/–^ mice, while septic JAM-A^–/–^ mice depleted of neutrophils lose their survival advantage. Therefore, increased bacterial clearance via neutrophils and an altered systemic inflammatory response with increased opsonizing IgA produced through the adaptive immune system results in improved survival in septic JAM-A^–/–^ mice. JAM-A may be a therapeutic target in sepsis via immune mechanisms not related to its role in permeability.

## Graphical abstract

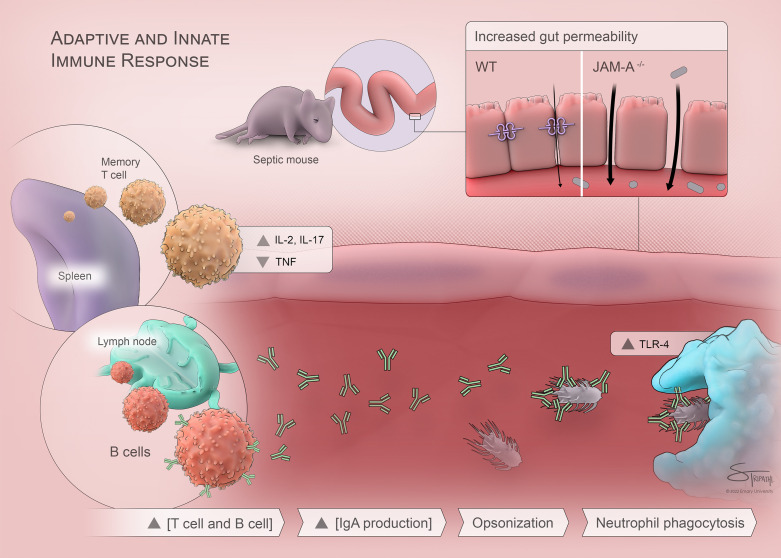

## Introduction

Sepsis is a life-threatening organ dysfunction caused by a dysregulated host response to infection and is responsible for nearly 20% of deaths worldwide (1, 2). In the United States, sepsis kills approximately 300,000 people annually (3), at a cost of >$62 billion for Medicare beneficiaries alone (4). Despite the morbidity and mortality associated with sepsis, no therapy is available to target the host response if antibiotics prove ineffective at controlling infection and the secondary systemic response (5).

The gut has long been hypothesized to be the motor of critical illness (6–9). A single-cell-layer intestinal epithelium functions as a physical barrier that separates the human host from the approximately 40 trillion microorganisms that reside in the gut microbiome (10). The gut barrier prevents intact bacteria and pathogenic components of the microbiome from entering the host while simultaneously allowing paracellular movement of water, solutes, and immune-modulating agents (11, 12). Sepsis induces intestinal hyperpermeability, allowing contents ordinarily restricted to the gut lumen to escape from their typical residence, which can secondarily alter the host inflammatory response and play a role in the propagation of systemic disease (13–16). The conversion of the gut microbiome to a pathobiome is associated with worsened outcomes in patients with sepsis and is directly mechanistically responsible for increased mortality in rodent sepsis (17–20).

Intestinal permeability is controlled in part via the apical tight junction (21). The tight junction mediates permeability via the pore pathway, a high-capacity, size- and charge-selective route through which only small molecules can pass, and the leak pathway, a low-capacity, nonselective route through which larger molecules such as LPS can pass (22). Notably, bacteria do not pass through either of these pathways but instead move via the unrestricted pathways, which are tight-junction independent and do not have a size limit.

Junctional adhesion molecule-A (JAM-A) is a transmembrane glycoprotein of the immunoglobulin superfamily that is expressed on the intestinal epithelium (23–25) and is a tight junction–associated PDZ binding domain (26). Germline deletion of JAM-A leads to increased intestinal permeability via the leak pathway, and epithelial cells lacking JAM-A have decreased transepithelial electrical resistance and enhanced paracellular flux in vitro (27, 28). The mechanisms through which JAM-A affects permeability to both small molecules and larger molecules are complex and appear to be mediated via association with ZO-2, afadin, and PDZ-GEF1 to activate Rap2c and control contraction of the apical cytoskeleton (29, 30). Notably, JAM-A levels are elevated in the intestinal epithelium, according to multiple different models of mouse sepsis (13). In addition to its expression on the intestinal epithelium, JAM-A is also expressed on a variety of leukocytes (including lymphocytes, neutrophils, monocytes, and dendritic cells) and endothelial cells (31, 32), where a variety of different functions have been described (33, 34). Notably, alterations in intestinal permeability in mice with intestine-specific deletion of JAM-A impair neutrophil recruitment after an acute inflammatory insult with either LPS or zymosan, and this is not seen in mice with leukocyte deletion of JAM-A in the same models (23). Given (1) the proposed role of gut hyperpermeability in mediating the host immune response and mortality from sepsis, and (2) JAM-A’s role in mediating gut permeability and immune function, we sought to determine the role of JAM-A deletion in a mouse model of polymicrobial sepsis.

## Results

### Septic JAM-A^–/–^ mice have increased intestinal permeability.

**Figure 1:**
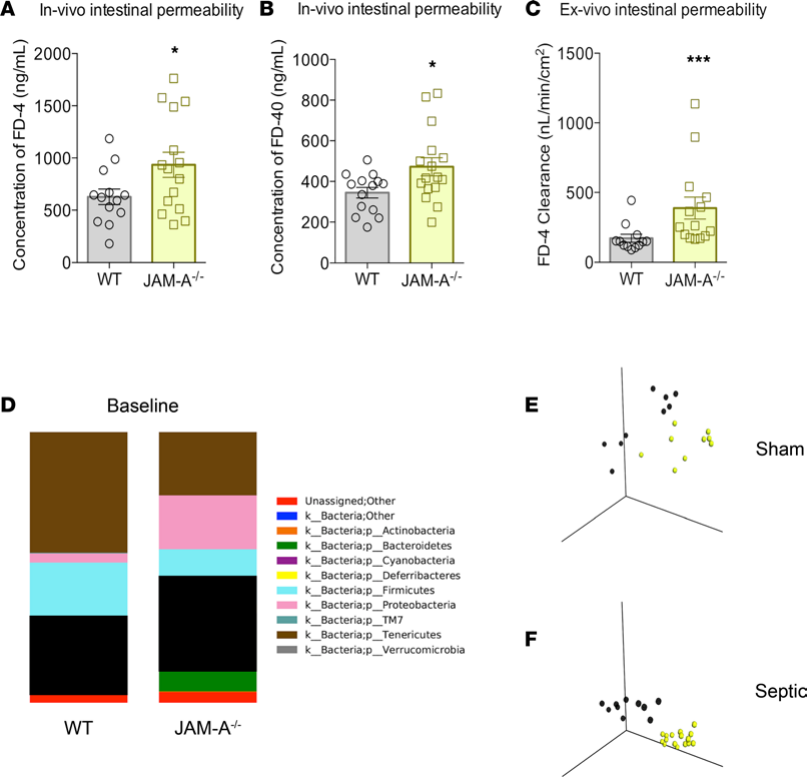
Intestinal permeability and microbiome in septic JAM-A^–/–^ mice. (**A** and **B**) Concentration of both FD-4 (*P* = 0.04; *n* = 13 WT, *n* = 15 JAM-A^–/–^) (**A**) and FD-40 (*P* = 0.02; *n* = 14 WT, *n* = 16 JAM-A^–/–^) (**B**) in the bloodstream was higher in septic JAM-A^–/–^ mice than in WT mice after oral gavage. (**C**) Permeability to FD4 was also higher ex vivo in the jejunum in an everted gut sac in JAM-A^–/–^ mice (*P =* 0.0008; *n =* 12 WT, *n =* 14 JAM-A^–/–^). (**D**) WT mice had different baseline phyla: Deferribacteres (*P =* 0.0004), Proteobacteria (*P =* 0.008), and Tenericutes (*P* = 0.008) than JAM-A ^–/–^ mice (*n* = 9 WT, *n* = 9 JAM-A^–/–^). (**E** and **F**) Baseline β diversity (between group differences) was statistically different between WT and JAM-A^–/–^ mice at baseline (*P* = 0.001; *n* = 9 WT, *n* = 9 JAM-A^–/–^) and after CLP (*P* = 0.001; *n* = 10 WT, *n* = 16 JAM-A^–/–^). (**A**–**C**) Data were subjected to a 1-tailed *t* test and the Mann-Whitney *U* test depending on presence of Gaussian distribution. (**E** and **F**) Data were subjected to ANOSIM test. *P < 0.05, **P < 0.01, ***P < 0.001, ****P < 0.0001.

Intestinal permeability was greater in septic JAM-A^–/–^ mice compared with WT mice, with increasing FD-4 appearance in the blood 5 hours after oral gavage (Figure 1A). Intestinal permeability to the larger molecule FD-40 was also increased in JAM-A^–/–^ mice (Figure 1B). These findings demonstrate that the gut barrier is compromised in JAM-A^–/–^ mice but cannot discriminate which portion of the intestine has hyperpermeability. To do so, permeability was measured ex vivo using an everted gut sac. Permeability was increased to FD-4 in the jejunum of septic JAM-A^–/–^ mice compared with WT septic mice, demonstrating worsened barrier function in the small intestine (Figure 1C).

### Septic JAM-A^–/–^ mice have decreased bacteremia and systemic inflammation and improved survival.

Increased intestinal permeability is associated with the passage of both inflammatory mediators and intact bacteria into the extraluminal space, causing distant organ damage and widespread inflammation. Because the microbiome may play a role in propagating the dysregulated host response after sepsis-induced hyperpermeability, we compared the microbiome in WT and JAM-A^–/–^ mice. The microbiome was different under basal conditions and these differences persisted after the onset of sepsis (Figure 1, D–F).

**Figure 2:**
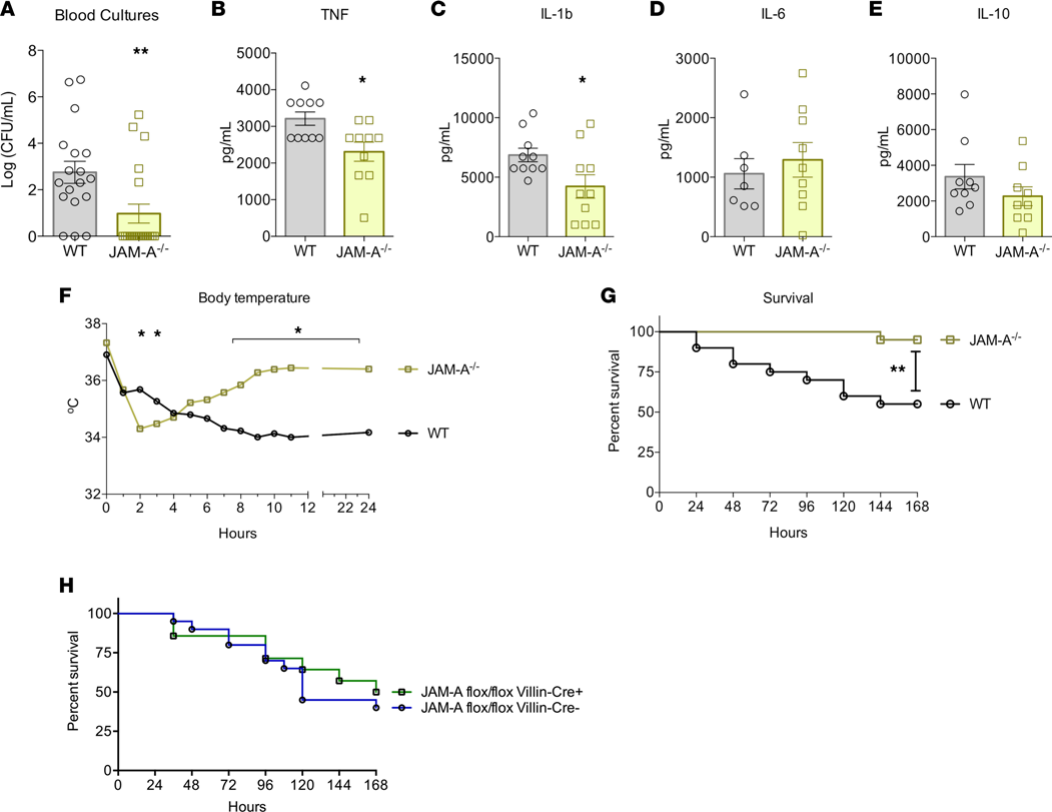
Bacteremia, cytokines, and survival in septic JAM-A^–/–^ mice. (**A**) Despite intestinal hyperpermeability, bacteremia was lower in JAM-A^–/–^ mice (*P =* 0.004; *n =* 18 WT, *n =* 20 JAM-A^–/–^); 75% of animals had no bacterial growth. (**B**–**E**) Serum levels of pro-inflammatory cytokines TNF (*P* = 0.01; *n* = 10 WT, *n* = 10 JAM-A^–/–^) and IL-1β (*P* = 0.03; *n* = 10 WT, *n* = 10 JAM-A^–/–^) were lower in septic JAM-A^–/–^ mice than in WT mice, whereas there were no differences in either IL-6 (*P* = 0.75; *n* = 10 WT, *n* = 10 JAM-A^–/–^) or IL-10 (*P* = 0.22; *n* = 9 WT, *n* = 9 JAM-A^–/–^) levels. (**F**) Body temperature was lower in septic JAM-A^–/–^ mice in the first 3 hours after sepsis. However, body temperature rapidly recovered in KO mice, whereas WT mice progressed to worsening hypothermia, which did not recover by 24 hours (*P* < 0.05 at 2, 3, 8, 9, 10, 11, and 24 hours; *P* > 0.05 at baseline and at 1, 4, 5, 6, and 7 hours; *n* = 5 WT, *n* = 7 JAM-A^–/–^ for each time point). (**G**) Survival rate was significantly higher in septic JAM-A^–/–^ mice (*P* = 0.003; *n* = 20 WT, *n* = 20 JAM-A^–/–^). (**H**) Survival was similar in mice with enterocyte-specific JAM-A deletion and WT mice (*P* = 0.28; *n* = 20 WT, *n* = 14 JAM-A^–/–^). (**A**–**F**) Data were subjected to a 1-tailed *t* testand the Mann-Whitney *U* test depending on presence of Gaussian distribution. (**G** and **H**) Log-rank test was applied to the data. *P < 0.05, **P < 0.01.

To determine if hyperpermeability through the leak pathway led to changes in bacterial burden, blood cultures were assayed in WT and JAM-A^–/–^ mice. Unexpectedly, blood cultures demonstrated markedly decreased bacterial burden in septic JAM-A^–/–^ mice, with 75% of KO mice having no bacterial growth (Figure 2A). In addition, septic JAM-A^–/–^ mice had decreased systemic levels of pro-inflammatory cytokines TNF and IL-1β (Figure 2, B and C) without alterations in IL-6 or IL-10 levels (Figure 2, D and E).

To determine whether changes in inflammation were associated with alterations in thermoregulation, KO and WT mice were assayed for body temperature hourly after the onset of sepsis (Figure 2F). Although JAM-A^–/–^ mice transiently had a lower temperature after sepsis, and by 4 hours their temperature matched that of the WT mice. After this, temperature curves diverged with worsening hypothermia in WT mice and normalization of body temperature in JAM-A^–/–^ mice, with differences persisting to 24 hours. Given the findings of increased intestinal permeability yet decreased systemic inflammation and bacterial burden and improved thermoregulation, we examined the impact of JAM-A deletion on mortality due to sepsis. Survival was significantly improved in JAM-A^–/–^ septic mice compared with WT septic mice (Figure 2G).

### Intestinal epithelial expression of JAM-A is not responsible for the survival benefit conferred in septic JAM-A^–/–^ mice.

In light of the association between intestinal permeability and apoptosis (35), we determined whether gut epithelial apoptosis was altered in JAM-A^–/–^ septic mice; we found no difference between WT and KO mice (Supplemental Figure 1, A and B; supplemental material available online with this article; https://doi.org/10.1172/jci.insight.156255DS1). No difference was noted in crypt proliferation in JAM-A^–/–^ septic mice either (Supplemental Figure 1C). Neither of these findings, however, rules out altered intestinal permeability as being causative in the improved mortality in JAM-A^–/–^ septic mice. Thus, based on the close relationship between intestinal permeability and mortality in sepsis, we next determined whether deleting JAM-A specifically in the intestinal epithelium was responsible for the mortality benefit seen in KO mice, given that mice with enterocyte-specific deletion of JAM-A have intestinal hyperpermeability compared with WT mice (12, 23, 25, 36). Enterocyte-specific JAM-A^–/–^ septic mice had similar mortality to WT mice, demonstrating JAM-A expression in the intestinal epithelium is not sufficient for the survival benefit seen in JAM-A^–/–^ septic mice (Figure 2H).

### Septic JAM-A^–/–^ mice have an increased percentage of CD44^hi^CD4^+^ T cells with decreased frequency of TNF^+^CD4^+^ cells and elevated frequency of IL-2^+^CD4^+^ cells.

**Figure 3:**
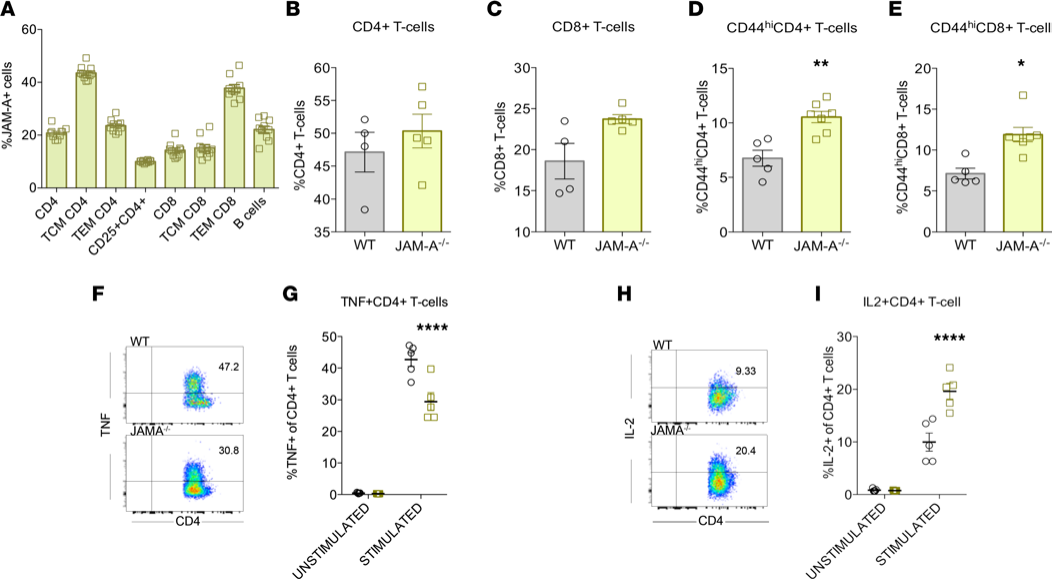
T cells in septic JAM-A^–/–^ mice. (**A**) JAM-A was expressed on CD4^+^ T cells, CD8^+^ T cells, and B cells in unmanipulated mice (*n* = 10 WT, *n* = 10 JAM-A^–/–^). (**B** and **C**) No statistically significant difference was noted in percentage of either CD4^+^ T cells (*P* = 0.52; *n* = 4 WT, *n* = 5 JAM-A^–/–^) or CD8^+^ T cells (*P* = 0.08; *n* = 4 WT, *n* = 5 JAM-A^–/–^) in the spleen. (**D** and **E**) In contrast, an increase was noted in the percentage of both memory CD44^hi^CD4^+^ T cells (*P* = 0.005; *n* = 5 WT, *n* = 7 JAM-A^–/–^) and memory CD44^hi^CD8^+^ T cells (*P* = 0.005; *n* = 5 WT, *n* = 7 JAM-A^–/–^) in septic JAM-A^–/–^ mice. (**F**–**I**) Splenocytes from septic JAM-A^–/–^ mice stimulated with PMA/ionomycin had decreased frequency of TNF^+^CD4^+^ cells (representative flow plot; *P* = 0.002; *n* = 5 unstimulated and 5 stimulated WT; *n* = 5 unstimulated and 5 stimulated JAM-A^–/–^) and increased frequency of IL-2^+^CD4^+^ cells (representative flow plot; *P* = <0001; *n* = 5 unstimulated and 5 stimulated WT; *n* = 5 unstimulated and 5 stimulated JAM-A^–/–^). (**B**–**E**, **G**, and **I**) Data were subjected to the Mann-Whitney *U* test. *P < 0.05, **P < 0.01, ****P < 0.0001.

Because JAM-A deletion was associated with changes in both inflammation and bacterial clearance, we examined its role in the immune system. A screen of unmanipulated WT mice demonstrated JAM-A is widely expressed in the adaptive immune system (Figure 3A). Based on this, the adaptive immune system was compared between septic WT and JAM-A^–/–^ mice. No differences in the percentage of CD4^+^ T cells or CD8^+^ T cells were detected in the spleen of septic WT and JAM-A^–/–^ mice (Figure 3, B and C). However, there was a significant increase in the percentage of memory CD44^hi^CD4^+^ T cells and memory CD44^hi^CD8^+^ T cells in septic JAM-A^–/–^ mice (Figure 3, D and E). Notably, this increase was specific to sepsis, because there were no significant differences in the percentages of memory CD44^hi^CD4^+^ T cells and memory CD44^hi^CD8^+^ T cells in sham WT and JAM-A^–/–^ mice (Supplemental Figure 2, A and B). To determine whether the differences in adaptive immune system changed cytokine production on a cellular level, splenocytes were stimulated with PMA/ionomycin. Stimulated splenocytes from septic JAM-A^–/–^ mice had decreased frequency of TNF^+^CD4^+^ cells (Figure 3, F and G) and elevated frequency of IL-2^+^CD4^+^ cells (Figure 3, H and I), both of which could alter antimicrobial response and potentially play a causative role in improved survival from sepsis. Of note, similar to the absence of changes in the percentage of bulk CD4^+^ T cells or CD8^+^ T cells in the spleen, no differences were identified in either mesenteric lymph nodes (MLNs) or Peyer’s patches in septic JAM-A^–/–^ mice (Supplemental Figure 3, A–D).

### Septic JAM-A^–/–^ mice have increased levels of serum and mucosal IgA.

**Figure 4:**
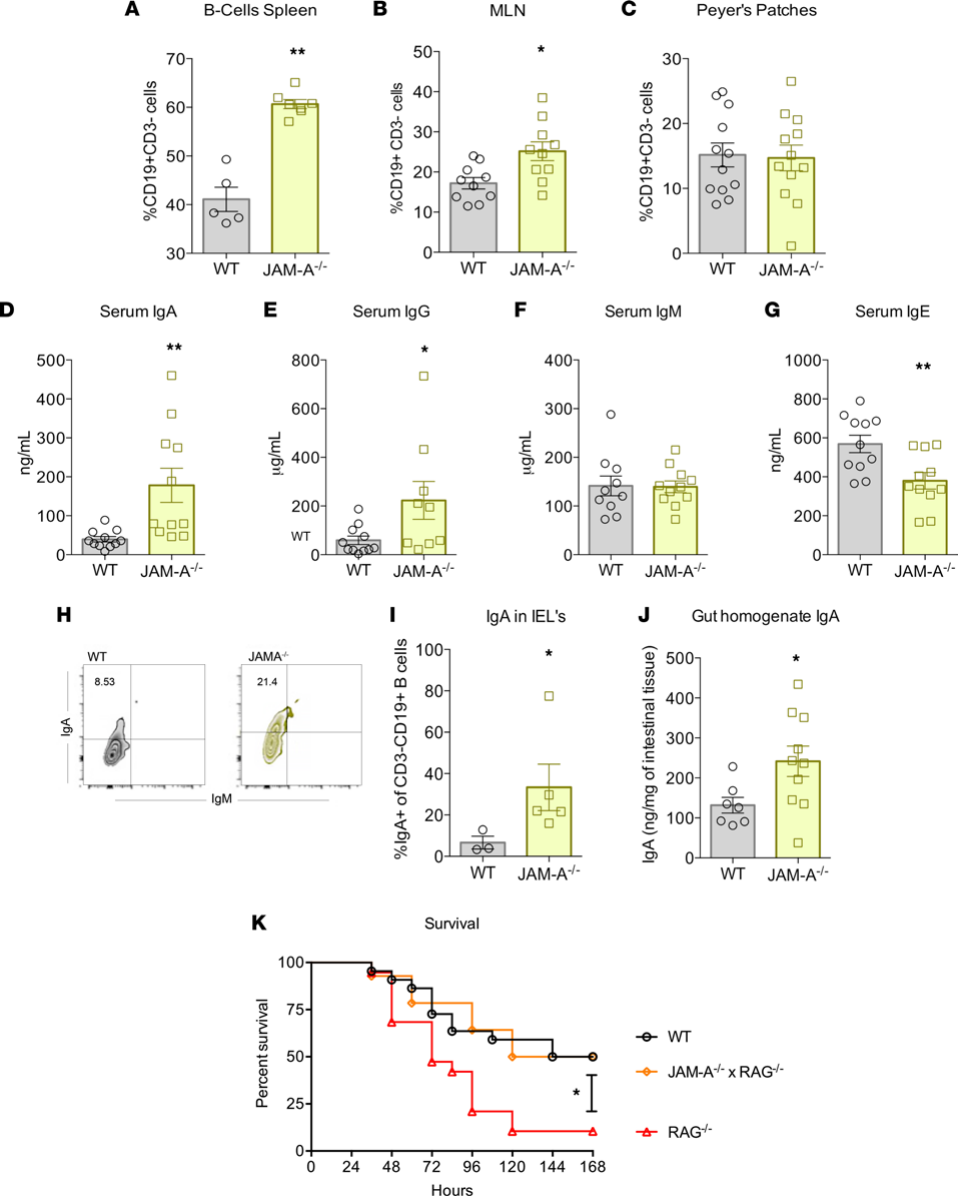
B cells and immunoglobulins in septic JAM-A^–/–^ mice. (**A**–**C**) Percentage of B cells increased in both spleen (*P* = 0.002; *n* = 5 WT, *n* = 7 JAM-A^–/–^) and MLNs (*P* = 0.009; *n* = 10 WT, *n* = 10 JAM-A^–/–^) in KO mice but were similar in Peyer’s patches (*P* = 0.86; *n* = 12 WT, *n* = 12 JAM-A^–/–^). (**D**–**G**) Levels of serum IgA (*P* = 0.005; *n* = 12 WT, *n* = 12 JAM-A^–/–^) and IgG (*P* = 0.02; *n* = 11 WT, *n* = 9 JAM-A^–/–^) were both higher in septic JAM-A^–/–^ mice, whereas IgM levels (*P* = 0.79; *n* = 10 WT, *n* = 11 JAM-A^–/–^) were similar and IgE levels (*P* = 0.006; *n* = 11 WT, *n* = 11 JAM-A^–/–^) were lower compared with WT mice. (**H** and **I**) Flow cytometry demonstrated increased frequency of IgA in intraepithelial lymphocytes (IELs) (representative flow plot; *P* = 0.04; *n* = 3 WT, *n* = 5 JAM-A^–/–^). (**J**) IgA levels also were increased in overall gut homogenate (*P* = 0.02; *n* = 7 WT, *n* = 10 JAM-A^–/–^). (**K**) Septic JAM-A^–/–^ × RAG^–/–^ mice had significantly improved survival compared with RAG^–/–^ mice, although survival was similar between JAM-A^–/–^ × RAG^–/–^ mice and WT mice, despite the latter having worse survival than JAM-A^–/–^ mice, as shown in Figure 2G (RAG^–/–^: *P* = 0.02, *n* = 22 WT, *n* = 19; JAM-A^–/–^ × RAG^–/–^: *n* = 14 JAM-A^–/–^ × RAG^–/–)^. (**A**–**G**, **I**, and **J**) Data were subjected to a 1-tailed *t* testand Mann-Whitney *U* test depending on presence of Gaussian distribution. (**K**) Log-rank test was applied to the data. *P < 0.05, **P < 0.01.

We next examined the frequency of B cells in both the local and systemic immune systems. B cell frequency increased in both the spleen and MLNs in septic JAM-A^–/–^ mice but was unchanged in Peyer’s patches (Figure 4, A–C). Given this increase of B cells, we sought to characterize their function in JAM-A^–/–^ animals. Because B cells produce immunoglobulins, quantification of serum immunoglobulins was performed. Serum IgA and IgG levels were significantly increased in septic JAM-A^–/–^ mice (Figure 4, D and E). No statistically significant differences were detected under sham conditions between WT and JAM-A^–/–^ mice for IgA levels (Supplemental Figure 4A), suggesting the increase in IgA seen in KO mice was sepsis specific. In contrast, serum IgG levels were increased under sham conditions in JAM-A^–/–^ mice in a manner similar to sepsis (Supplemental Figure 4B), revealing this is a manifestation of JAM-A deletion independent of sepsis. No differences in serum IgM were found between septic WT and JAM-A^–/–^ mice (Figure 4F), whereas IgE levels were lower in septic JAM-A^–/–^ mice (Figure 4G).

Based on the selective increase in serum IgA in septic JAM-A^–/–^ mice, evaluation of intraepithelial lymphocytes was performed to further define this response at a mucosal level. Flow cytometric analysis of B cells within the gastrointestinal lining revealed significantly increased IgA production in JAM-A^–/–^ septic mice compared with WT septic mice (Figure 4, H and I). This was confirmed via ELISA of gut homogenate tissue, revealing elevated IgA in septic JAM-A^–/–^ mice (Figure 4J).

To determine if the changes identified in the adaptive immune system in septic JAM-A^–/–^ mice were responsible for their improved survival in sepsis, JAM-A^–/–^ mice were crossed with RAG^–/–^ mice, which lack mature T and B lymphocytes. If the survival advantage was secondary to deletion of JAM-A in lymphocytes, we would anticipate that these double KO mice would have a similar sepsis mortality compared with RAG controls. However, septic JAM-A^–/–^ × RAG^–/–^ mice exhibited improved sepsis survival compared with septic RAG^–/–^ mice (Figure 4K), although septic JAM-A^–/–^ × RAG^–/–^ mice had the identical mortality as septic WT mice (Figure 4K), which, as shown earlier, was significantly lower than in septic JAM-A^–/–^ mice (Figure 2G). These finding demonstrates that lymphocyte expression of JAM-A is not solely responsible for the mortality benefit conferred in KO mice and that the adaptive immune system plays some role in sepsis mortality.

### Septic JAM-A^–/–^ mice have enhanced neutrophil bacterial phagocytosis, and depletion of neutrophils leads to abrogation of survival advantage.

**Figure 5:**
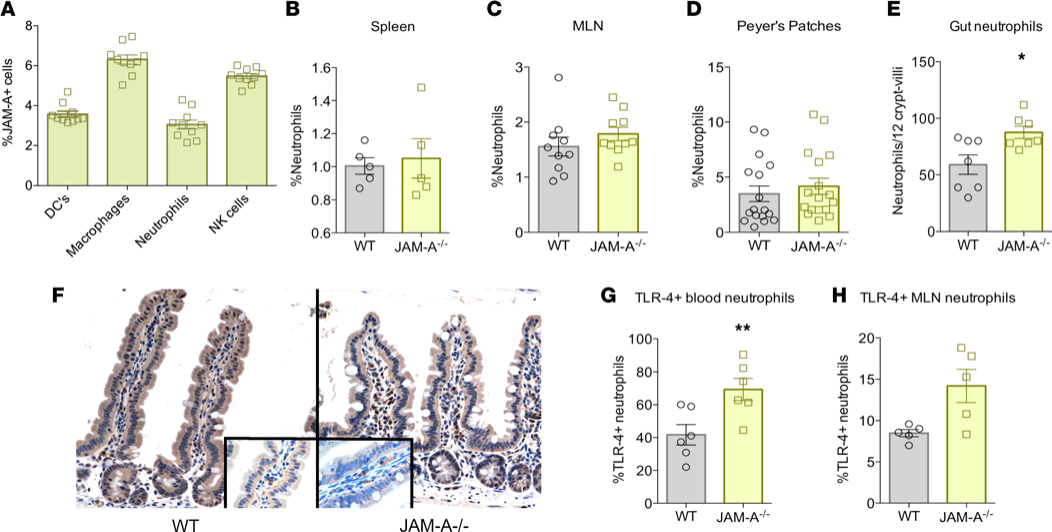
Neutrophils in septic JAM-A^–/–^ mice. (**A**) JAM-A was expressed on DCs, macrophages, neutrophils, and NK cells in unmanipulated mice (*n* = 10 WT, *n* = 10 JAM-A^–/–^). (**B**–**D**) Neutrophil frequency was similar between septic JAM-A^–/–^ and WT mice in spleen (*P* = 0.88; *n* = 5 WT, *n* = 5 JAM-A^–/–^), MLNs (*P* = 0.94; *n* = 10 WT, *n* = 11 JAM-A^–/–^), and Peyer’s patches (*P* = 0.50; *n* = 17 WT, *n* = 17 JAM-A^–/–^). (**E** and **F**) IHC staining for Ly6G cells in the intestine demonstrated an increase in septic JAM-A^–/–^ mice (*P* = 0.049; *n* = 7 WT, *n* = 8 JAM-A^–/–^). Representative histomicrographs are shown (cells in lamina propria stain brown; magnification: ×20 in main panel, ×40 in inset). (**G** and **H**) The percentage of TLR4^+^ neutrophils increased in the blood of septic JAM-A^–/–^ mice (*P* = 0.009; *n* = 6 WT, *n* = 6 JAM-A^–/–^), and a trend toward an increase was also noted in MLNs (*P* = 0.055; *n* = 5 WT, *n* = 5 JAM-A^–/–^). (**A**–**E**, **G**, and **H**) Data were subjected to a 1-tailed *t* test and Mann-Whitney *U* test for depending on presence of Gaussian distribution. *P < 0.05, **P < 0.01.

On the basis of changes in inflammation and bacterial clearance, we next examined the innate immune system. A screen of unmanipulated WT mice demonstrated JAM-A is widely expressed at low levels in the innate immune system (Figure 5A). No differences were detected in frequency of DCs or NK cells in the spleen or MLNs (Supplemental Figure 5, A–D). There were also no differences in percent macrophages in spleen and Peyer’s patches, although a slight percent increase was noted in MLNs of JAM-A^–/–^ mice (Supplemental Figure 6, A–C). Monocytes were also similar in Peyer’s patches but were slightly decreased in MLNs of JAM-A^–/–^ mice (Supplemental Figure 6, D and E). Although the frequency of neutrophils did not differ between septic WT and JAM-A^–/–^ mice (Figure 5, B–D), there was increased neutrophil infiltration in the gut as measured by Ly6G^+^ cells in septic JAM-A^–/–^ mice (Figure 5, E and F). Analysis of innate cellular activation also revealed an increase in the percentage of TLR4^+^ neutrophils in the blood of septic JAM-A^–/–^ mice (Figure 5G) as well as a trend toward an increase (*P =* 0.055) in the percentage of TLR4^+^ neutrophils in MLNs (Figure 5H), suggesting that circulating neutrophils may be more activated or primed to clear invasive bacteria.

**Figure 6:**
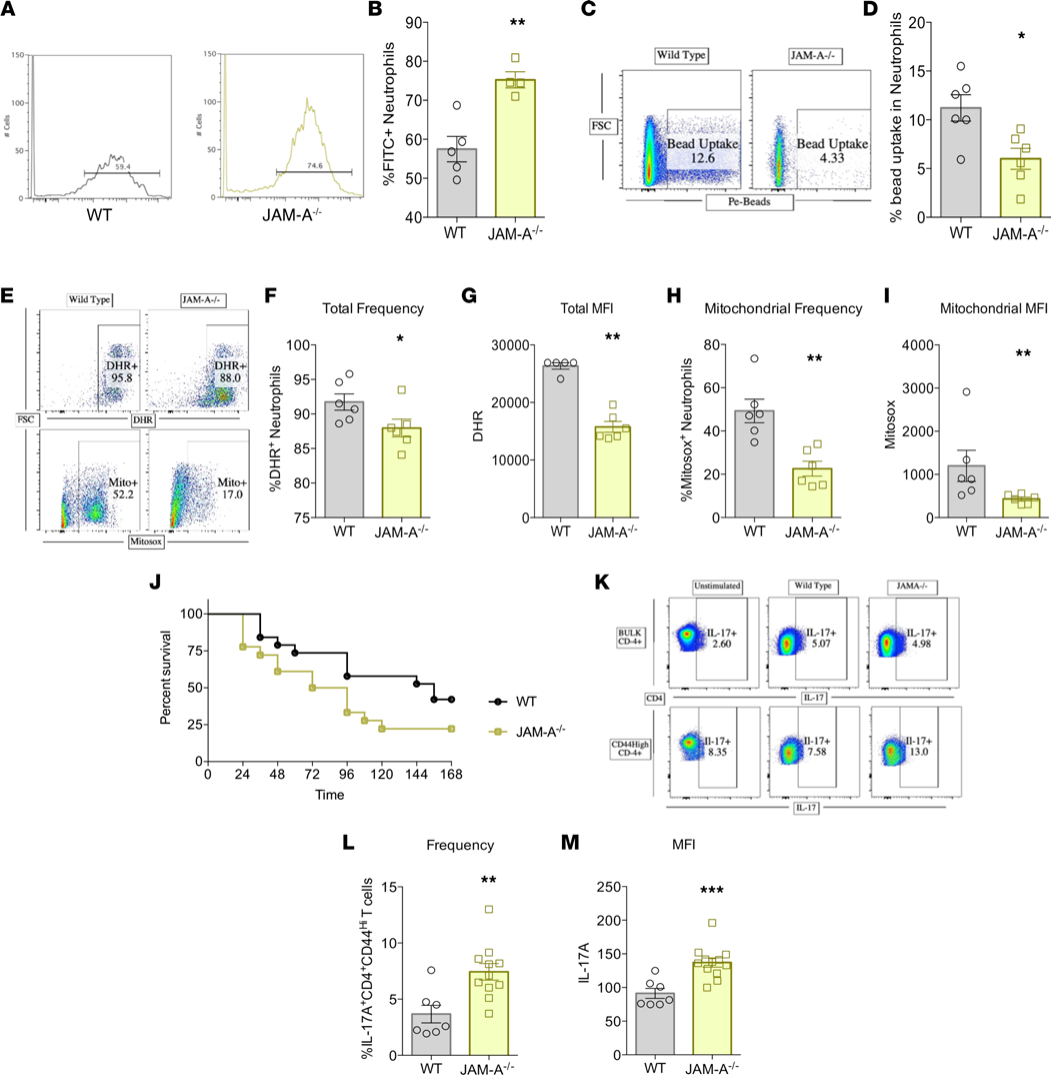
Phagocytosis, ROS production, and survival in septic JAM-A^–/–^ mice depleted of neutrophils. (**A** and **B**) Neutrophil phagocytosis was increased to fluorescently tagged *E*. *coli* in septic JAM-A^–/–^ mice (representative experiment; *P* = 0.01; *n* = 5 WT, *n* = 6 JAM-A^–/–^). (**C** and **D**) In contrast, neutrophil phagocytosis was decreased to fluorescently tagged inert PE beads in septic JAM-A^–/–^ mice (representative flow plot; *P* = 0.015; *n* = 9 WT, *n* = 6 JAM-A^–/–^). (**E**–**I**) JAM-A^–/–^ mice had decreased total (representative flow plot, frequency: *P* = 0.03; MFI: *P* = 0.009, *n* = 6 WT, *n* = 6 JAM-A^–/–^) and mitochondrial (representative flow plot, frequency: *P* = 0.002; MFI: *P* = 0.009, *n* = 6 WT, *n* = 6 JAM-A^–/–^) ROS compared with WT mice. (**J**) In contrast to immunocompetent septic JAM-A^–/–^ mice, which had improved survival compared with WT mice (Figure 2G), septic JAM-A^–/–^ mice depleted of neutrophils lost their survival advantage and had a trend toward increased mortality (*P* = 0.009; *n* = 19 WT, *n* = 18 JAM-A^–/–^). (**K**–**M**) Frequency of splenic IL-17^+^ cells in memory CD4+ cells was higher in septic JAM-A^–/–^ mice (representative flow plot, frequency: *P* = 0.003; MFI: *P* = 0.0008, *n* = 7 WT, *n* = 12 JAM-A^–/–^). (**B**, **D**, **F**–**I**, **L**, and **M**) Data were subjected to Mann-Whitney *U* test. (*J*) Data were subjected to a log-rank test. *P < 0.05, **P < 0.01, ***P < 0.001.

Because innate cell functionality includes bacterial phagocytosis, we evaluated functionality of circulating neutrophils via a phagocytosis assay. Quantification of Gr1^+^ cells that were positive for uptake of fluorescently tagged *E*. *coli* showed that septic JAM-A^–/–^ mice exhibited an increase in neutrophil phagocytosis compared with WT septic mice (Figure 6, A and B). This change in neutrophil phagocytosis is specific for bacterial phagocytosis because JAM-A^–/–^ mice had decreased neutrophil phagocytosis to inert beads, compared with WT septic mice (Figure 6, C and D). Despite the increased phagocytosis to *E*. *coli,* JAM-A^–/–^ mice had decreased total and mitochondrial ROS production compared with that of WT mice (Figure 6, E–I).

On the basis of these disparate results, we examined whether the survival advantage in septic JAM-A^–/–^ mice was dependent upon the presence of neutrophils. A Ly6G-depleting Ab was given to WT and JAM-A^–/–^ mice 24 hours prior to cecal ligation and puncture (CLP) (Supplemental Figure 7, A and B), and survival was compared. Whereas septic JAM-A^–/–^ mice had improved survival compared with septic WT mice (Figure 2G), septic JAM-A^–/–^ mice depleted of neutrophils lost their survival advantage compared with septic WT mice and had a trend toward worsened 7-day mortality (Figure 6J). This demonstrates the importance of neutrophils in mediating the survival advantage seen in septic JAM-A^–/–^ mice.

Finally, because IL-17 produced by CD4^+^ cells has been shown to affect neutrophil migration, we examined production of this cytokine in septic JAM-A^–/–^ mice. Although no difference in the frequency of Il-17^+^ was detected on bulk splenic CD4^+^ cells (Supplemental Figure 8, A and B), there was a significant increase in the frequency of IL-17^+^ cells in memory splenic CD4^+^ cells (Figure 6, K–M).

## Discussion

This study demonstrates that despite having increased intestinal permeability, septic JAM-A^–/–^ animals have a marked decrease in bacteremia and improved mortality after sepsis. Mechanisms underlying improved survival include increased bacterial clearance via increased neutrophil phagocytic capacity in the innate immune system and increased IgA production along with elevated frequency of IL-2^+^CD4^+^ cells in the adaptive immune system. Notably, these differences were not sex based: the differences identified between JAM-A^–/–^ and WT mice were seen in both male and female mice.

The hypothesis of gut-derived sepsis is >30 years old and conceptually states that increased permeability to both intestinal microbes and microbial products leads to systemic inflammation as well as bacteremia and, ultimately, death (37). The gut barrier is mediated via tight junctions that alter paracellular movement via 2 distinct pathways: a high-capacity, size- and charge-selective route (pore pathway) and a low-capacity, nonselective route (leak pathway) (22). Only small molecules can pass through the pore pathway (<8 Å), whereas larger molecules can pass via the leak pathway (<100 Å) (38). In addition, a third tight junction–independent pathway of permeability (unrestricted pathway) that does not have a size limit occurs at sites of epithelial damage and apoptosis, and bacteria can only cross the epithelium via this pathway. On the basis of JAM-A’s known role in mediating intestinal permeability via the leak pathway, our pre hoc hypothesis was that septic JAM-A^–/–^ mice would have increased permeability to large molecules, leading to increased inflammation and mortality. It was difficult to predict a priori how bacteremia would be affected in JAM-A^–/–^ mice. In theory, it would be plausible that increased passage of large molecules from the gut lumen would lead to increased inflammation, which, in turn, would cause increased epithelial damage and apoptosis, allowing increased bacterial translocation via the unrestricted pathway and increased bacteremia. It would also have been plausible that bacteremia would be unchanged because JAM-A mediates the leak pathway but not the unrestricted pathway. However, neither of these turned out to be correct. Although septic JAM-A^–/–^ mice had increased gut permeability through the leak pathway as expected, inflammation, bacteremia, and mortality all decreased. This finding suggests that the decrease in bacteremia and inflammation we identified was mediated by an effect of JAM-A on other cell types that is either independent of gut leak permeability or sufficient to overcome increased gut leak.

The relationship between gut permeability and mortality in sepsis is significantly more complex than originally understood (6, 7, 39–42), especially because in most published research examining intestinal permeability in critical illness, a single agent was used that cannot distinguish among the 3 pathways of permeability. The unexpected findings in mice with a germline deletion of JAM-A led us to examine mice with gut-specific JAM-A deletion to help clarify why bacteremia was seemingly paradoxically decreased in mice with worsened permeability via the leak pathway. Our data showing no difference in survival in these mice compared with WT mice lead to the conclusion that the beneficial impact of JAM-A germline deletion is due to cells outside of the gut epithelium. Notably, this does not negate the concept that bacterial translocation is important in the pathophysiology of sepsis but rather the highlights the need to examine both (1) size-specific permeability in the gut via the 3 distinct pathways of permeability, and (2) extra-intestinal effects, even for molecules known to mediate processes within the gut epithelium.

In addition to its role in intestinal permeability, JAM-A is also expressed on multiple different immune cells (Figure 3A and Figure 5A) where it plays a wide variety of physiologic roles (31, 32). On the basis of the critical role the adaptive immune system plays in controlling infection in sepsis and modulating the host inflammatory response (43, 44), we examined the role of JAM-A on lymphocytes in sepsis. Previous studies demonstrated that treatment with either anti–JAM-A Ab or JAM-A–Fc fusion protein inhibits migration of human memory CD4^+^ T cells (45). Notably, we identified a significant sepsis-specific increase in memory CD44^hi^CD4^+^ T cells in JAM-A^–/–^ mice that was not present in sham mice. Furthermore, stimulated splenocytes from septic JAM-A^–/–^ mice had elevated frequency of IL-2^+^CD4^+^ T cells and decreased frequency of TNF^+^CD4^+^ T cells. An increase in IL-2–producing cells is associated with improved immune competence and mounting a productive antimicrobial response (46). Additionally, cells that produce IL-2 exist in a less terminally differentiated state, suggesting they are still under the control of several checkpoints and are less likely to be dysregulated (47). Furthermore, critical illness has been associated with suppression of IL-2, promoting a predominant Th2 environment, resulting in ineffective T cell responses (48–50). This suggests a potential role for IL-2 in the improved survival in JAM-A^–/–^ animals. In addition, considering the role of TNF in promoting ongoing deleterious inflammation in sepsis**,** decreased production is likely to be beneficial (51, 52). And TNF inhibits neutrophil migration to the peritoneum in response to polymicrobial sepsis by decreasing CXCR2 expression and inducing apoptosis (52).

Although a small body of literature exists on the relationship between both junctional adhesion molecule-B (53) and junctional adhesion molecule-C (54) and B cells, little is known about the role JAM-A plays on B cells. We identified an increase in the frequency of B cells in septic JAM-A^–/–^ animals, which was of potential significance in mediating their mortality benefit, considering the role of B cell deletion in chronic immunoparalysis after sepsis (55). This led us to examine immunoglobulins, where we identified a sepsis-specific increase in serum IgA in septic JAM-A^–/–^ mice. A similar increase in mucosal IgA was identified using flow cytometry in intraepithelial lymphocytes and when examining ELISA of gut homogenate tissue. IgA plays an integral role in maintaining homeostasis in the gut microbiota, with low-affinity IgA contributing to microbiota maintenance and high-affinity IgA acting on pathogens to promote clearance and inhibit virulence (56, 57). There is an important relationship between JAM-A and IgA in terms of susceptibility to colitis. Whereas IgA deletion does not affect disease in WT mice, mice with simultaneous deletion of both JAM-A and IgA have a markedly increased susceptibility to dextran sulfate sodium–induced acute colitis (28). Lower IgA levels are also associated with increased mortality in human septic shock (58, 59). Furthermore, IgA induction protects against lethal sepsis after intestinal barrier disruption in mice (60).

In addition, we identified a decrease in IgE levels in septic JAM-A^–/–^ mice. It is notable that high IgE levels at admission predict increased mortality in patients presenting to the emergency department with sepsis, and it is possible that IgE plays a role in mediating the mortality difference identified in JAM-A^–/–^ mice, although we cannot conclude causation from this associative finding (61). Together, these findings in both T cells and B cells suggested plausible mechanisms through which the adaptive system may have been responsible for both control of bacterial burden and the survival benefit seen in mice with genetic deletion of JAM-A.

However, the mortality studies in JAM-A^–/–^ × RAG^–/–^ mice yielded complicated results. If the survival benefit seen in septic JAM-A^–/–^ mice was solely due to lymphocytes, mortality should have been similar between double KOs and RAG^–/–^ mice. The finding that septic JAM-A^–/–^ × RAG^–/–^ mice had higher survival rates after CLP than did RAG^–/–^ mice suggests that mortality must be at least partially lymphocyte independent. However, that septic JAM-A^–/–^ × RAG^–/–^ mice have similar mortality as WT mice (which, in turn, have higher mortality rates than JAM-A^–/–^ mice) suggests the adaptive immune system likely plays some role in mediating the survival benefit conferred by deletion of JAM-A. This is consistent with prior studies demonstrating that although lymphocyte deletion alone has negligible contribution to development of colitis, JAM-A^–/–^ × RAG^–/–^ mice have markedly increased susceptibility to microflora-dependent colitis (28).

These observations led to an examination of the innate immune system, given the robust literature on the relationship between JAM-A and neutrophils. Specifically, JAM-A is required for neutrophil infiltration in inflammatory or ischemic tissues, and JAM-A–deficient neutrophils have reduced diapedesis in peritonitis induced by thiogycollate (62, 63). JAM-A has a cell autonomous role in neutrophil chemotaxis and is required for correct internalization and recycling of integrins during cell migration (64). Furthermore, JAM-A^–/–^ mice have decreased neutrophil recruitment to the peritoneum after administration of zymosan, LPS, or TNF via a mechanism that is dependent on intestinal barrier function; similar findings were seen in mice with JAM-A deficiency in the intestinal epithelium (23). JAM-A^–/–^ mice also have increased neutrophil accumulation in the intestinal mucosa at baseline (36), similar to what was seen in this study in septic mice. Although neutrophil accumulation could potentially have led to decreased bacteremia in KO mice, this did not rule out a direct role for JAM-A in mediating bacterial clearance. As such, a phagocytosis assay for uptake of fluorescently tagged *E*. *coli* was performed, which demonstrated increased phagocytosis in septic JAM-A^–/–^ mice despite decreased total and mitochondrial reactive species production in the blood. The increased phagocytosis may be related to the increased TLR4 expression seen in neutrophils of JAM-A^–/–^ mice. To our knowledge, this is a novel finding because we are unaware of previous evidence linking JAM-A and phagocytosis. Furthermore, this finding was not due to a nonspecific increase in JAM-A^–/–^ mice, because they had decreased phagocytosis of inert beads, suggesting at least some degree of specificity to the response. To determine the functional significance of these findings, septic JAM-A^–/–^ mice depleted of neutrophils not only lost their survival advantage compared with septic WT mice but also trended toward a higher mortality rate. This indicates that neutrophils play a crucial role in mediating the improved outcomes seen in JAM-A^–/–^ mice. A potential explanation tying our results together is that increased gut leakiness leads to both increased IgA production and immune cell priming (23), resulting in a hyperresponsive phenotype in neutrophils. This suggests that changes in both the innate and adaptive immune systems induced by intestinal hyperpermeability are responsible for the altered mortality seen in JAM-A^–/–^ mice. To determine a potential link between the innate and adaptive immune systems, we examined IL-17A, because this is a potent inflammatory cytokine playing an essential role in granulopoiesis and neutrophil migration (65). We found an increase in IL-17^+^CD44^+^CD4^+^ T cells in JAM^–/–^ septic mice that was specific to the memory Th17 cell compartment, because it was not present in bulk CD4^+^ T cells. Notably, IL-17R–deficient mice have less neutrophil recruitment into the peritoneal cavity and increased mortality compared with WT mice after CLP (66). Although an increased proportion of Th17 cells has been shown to be associated with worse prognosis in patients with sepsis (67), we instead found that an increase in the number of memory Th17 cells in JAM^–/–^mice was associated with improved survival. It is possible that these cells play a role in neutrophil recruitment to limit infection; however, more experiments need to be performed to confirm this hypothesis.

This study has a number of limitations. First, JAM-A^–/–^ mice have a germline genetic deletion. This leads to all animals having a lifelong deficiency in JAM-A, which means that the impact of targeting JAM-A specifically after the onset of sepsis is unknown. Furthermore, because JAM-A has multiple effects on multiple cell types, it is possible that there are effects not measured in other cells where JAM-A is expressed, such as endothelial cells. Additionally, outside of survival studies, only a single time point was assayed. Although 24 hours was chosen because multiple elements of both gut and immune integrity are maximally altered in murine models of sepsis at this time (13, 68–70), it is likely that the lack of a time course prevents insights that can only be obtained with repeated sampling (71, 72). We also cannot rule out that the differences in the microbiome under both basal and septic conditions played a role in mediating mortality between WT and JAM^–/–^ mice, given the importance of the microbiome in mediating mortality in sepsis (17, 73). However, given that selective JAM-A deletion in the intestine did not alter mortality compared with WT mice, it seems less likely that the microbiome was primarily responsible for the survival advantage noted in septic JAM^–/–^ mice. Also, we did not examine cytokine concentrations (namely, IL-2 and TNF) in septic JAM-A^–/–^ × RAG^–/–^ versus RAG^–/–^ mice, which may have yielded additional insights.

Despite these limitations, these studies demonstrate a role for JAM-A in survival after sepsis, with alterations in both the innate and adaptive immune responses. Although the molecular mechanisms that determine how JAM-A modulates phagocytosis and IgA production in the setting of a polymicrobial infection remain to be determined, inhibiting JAM-A represents an unexpected strategy for the treatment of sepsis. More studies are required to determine the translational potential of targeting JAM-A.

## Methods

### Animals.

Experiments were performed on 6- to 12-week-old, sex-matched male and female mice. C57BL/6 WT and RAG^–/–^ mice were purchased from Jackson Laboratories. JAM-A^–/–^ mice and animals with intestine-specific deletion of JAM-A (Villin^cre^JAM-A^FL/FL^ mice) were created as previously described (74, 75). JAM-A^–/–^ × RAG^–/–^ mice were created by crossing JAM-A^–/–^ mice with RAG^–/–^ mice through to the F2 generation. Mice were maintained on a 12-hour light-dark schedule in a specific pathogen-free environment. All animals received standard laboratory mouse chow and water ad libitum at all times.

### Sepsis model.

Mice underwent CLP to induce polymicrobial i.p. sepsis. Surgery was performed under isoflurane anesthesia using aseptic technique. Through a small midline incision, the cecum was identified. The cecum was ligated at approximately 50% of its length without causing intestinal obstruction and was punctured twice with a 25G needle. A small amount of stool was extruded from the cecum before being returned to the abdomen, which was closed in layers. In a subset of experiments, core body temperature was measured hourly after CLP out to 12 hours and then at 24 hours by measuring back-skin temperature using an infrared thermometer (Braun Healthcare). To mimic clinical management (5), all mice received fluids and antibiotics. Specifically, animals received 1 mL of 0.9% NaCl via s.c. injection to account for insensible fluid losses (76) as well as s.c. injections of ceftriaxone (50 mg/kg; Sigma-Aldrich) and metronidazole (30 mg/kg; Apotex Corp.) immediately after CLP and every 12 hours after out to 48 hours (5). Animals were either euthanized at 24 hours after CLP for functional studies or followed for 7 days for survival studies. All mice received buprenorphine (0.1 mg/kg; McKesson Medical) preoperatively and were re-dosed as deemed appropriate by Emory University Division of Animal Resources postoperatively.

### Intestinal permeability.

Animals received an oral gavage of 0.5 mL of 22 mg/mL fluorescein isothiocyanate–conjugated dextran (either FD-4 or FD-40, molecular mass 4 KDa or 40 KDa; Sigma-Aldrich) 5 hours before sacrifice (19 hours after CLP). When animals were sacrificed, whole blood was collected and then centrifuged at 10,600*g* at 4°C for 10 minutes. An aliquot of plasma was diluted 1:2 in PBS and the concentration of the gavaged agent was determined using fluorospectrometry (Synergy HT, BioTek) at an excitation of 485 nm and emission of 528 nm. All samples were run in duplicate.

Ex vivo intestinal permeability was determined using the everted gut-sac method. Proximal small intestine was removed from the abdomen, flushed with ice-cold Krebs–Henseleit bicarbonate buffer (KHBB; pH 7.4) and then submerged in ice-cold KHBB. One end of the intestine was then ligated with 4-0 silk suture. The intestine was then everted over a glass rod. The gut sac was then filled with 1 mL of KHBB and placed in a container of 100 μg/mL FD-4 in KHBB for 30 minutes. The container was temperature jacketed to 37°C and gently aerated with 100% oxygen. After 30 minutes, the sac was removed and the fluid inside the sac was collected. The length and diameter of each sac were measured. The fluid was centrifuged at 10,600*g* for 10 minutes and the supernatant was collected and stored. The fluid was diluted 1:2 with PBS and the concentration of FD-4 was determined by fluorospectometry, as described above. Permeability was expressed as clearance of FD-4 from mucosa to serosa in nanoliters per minute per centimeter squared. All samples were run in duplicate.

### Microbiome analysis.

Colonic samples were collected at time of euthanasia and were frozen at –80°C until samples were sent to the Emory Genomics Core Laboratory where bacterial DNA was extracted. DNA was isolated using the Powersoil kit according to the manufacturer’s protocol (Qiagen). We prepared 16s rRN V3 and V4 libraries as outlined in the Illumina 16s-metagenomic library preparation guide (version 15044223-b). Indexed libraries were pooled and sequenced on an Illumina MiSeq using a 600 cycle V4 reagent kit (Illumina). The resulting raw sequences were quality controlled with default parameters, including eliminating inadequate sequences and chimeras, using the QIIME, version 1.9.1, software package (http://qiime.org/index.html). The resulting sequences were clustered using a closed-reference frame at 97% similarity against the GreenGenes database (version 13_8; https://greengenes.secondgenome.com/) to assign bacterial operational taxonomic units. Samples were rarified to a depth of 2000 copies. β diversity using unweighted UniFrac distances were then plotted on a principal coordinates analysis plot, using QIIME software (77).

### Blood culture.

Using sterile technique, whole-blood samples were collected with heparinized syringes via cardiac puncture and put into EDTA-lined tubes at the time of sacrifice. Blood samples were serially diluted and directly plated on sheep’s blood agar plates and incubated at 37°C in 5% CO_2_. After 24 hours, colony counts were measured.

### Serum cytokines.

Whole blood was collected and centrifuged at 10,600*g* for 10 minutes. Plasma cytokine concentrations were determined using a multiplex magnetic-bead cytokine assay kit according to the manufacturer’s protocol (Bio-Rad). All samples were run in duplicate.

### Intestinal IHC.

Jejunal epithelial apoptotic cells were quantified by 2 complementary methods: active caspase-3 staining and H&E staining. For active caspase-3, sections were deparaffinized, rehydrated, and incubated in 3% hydrogen peroxide for 10 minutes. Slides were then immersed in Antigen Decloaker (Biocare Medical) and heated in a pressure cooker for 45 minutes, then blocked with 20% goat serum (Vector Laboratories). Sections were then incubated with rabbit polyclonal anti–active caspase-3 (1:100; Cell Signaling Technology) overnight at 4°C. The next day, slides were incubated with goat anti-rabbit biotinylated secondary Ab (1:200; Vector Laboratories) for 30 minutes at room temperature, followed by Vectastain Elite ABC reagent (Vector Laboratories) for 30 minutes and developed with diaminobenzidine followed by hematoxylin counterstaining. For morphologic analysis, apoptotic cells were identified on H&E-stained sections using characteristic morphology including nuclear condensation and fragmentation.

To examine proliferation, mice received an i.p. injection of BrdU (50 mg/kg, diluted in 0.9% saline; Sigma) to label cells in S phase. BrdU was detected in jejunal sections via IHC as described above, except sections were blocked for 30 minutes with Protein Block (Dako) and then incubated with rat monoclonal anti-BrdU (1:500; Accurate Chemical & Scientific) overnight at 4°C and were then incubated with goat anti-rat secondary Ab (1:500; Accurate Chemical & Scientific), followed by streptavidin-HRP (1:500; Dako).

For Ly6G staining, sections were incubated with rat monoclonal anti-Ly6G (1:500; eBioscience Clone 1A8) overnight at 4°C. The following day, samples were incubated with goat anti-rat secondary Ab (1:200; Vector Laboratories), and otherwise were stained as above. Positive cells were quantified in IHC analysis by an observer blinded to sample identity (NJK).

### Phenotypic flow cytometric analysis and intracellular cytokine staining.

For phenotypic analysis, animals were sacrificed at 24 hours via CO_2_ euthanasia. Tissue harvested for examination included the spleen, MLNs, and Peyer’s patches. Each tissue was processed into single suspensions. The number of cells per milliliter of suspension was obtained via a Nexcelom Auto Cellometer. We plated 2 × 10^6^ cells onto a 96-well plate and stained them for extracellular markers. TruCount Beads (BD Pharmingen) were prepared according to the manufacturer’s instructions and used to determine absolute cell counts.

For cytokine analysis, 2 × 10^6^ splenic cells were plated onto a 96-well plate. Cells were suspended in RPMI 1640 culture medium and incubated for 4 hours at 37°C using PMA (30 ng/mL) and ionomycin (400 ng/mL) with 10 μg/mL Brefeldin A. After incubation, cells were stained with extracellular markers, processed with an intracellular staining kit (BD Biosciences) per manufacturer’s protocol, and stained for intracellular cytokine markers. TruCount Beads (BD Pharmingen) were prepared according to manufacturer’s instructions and used to determine absolute cell counts. Additional data on markers, conjugates, and clones used for flow cytometry are available in Supplemental Table 1. Data were acquired on an LSRII Flow cytometer (BD Biosciences) and subsequently analyzed with FlowJo 10.0.8rl software (Tree Star).

### Immunoglobulin ELISA.

A total of 1 μL of whole blood was centrifuged at 10,600*g* for 10 minutes to collect serum supernatant. In addition, for gut-homogenate assay and protein extraction, small segments of small intestine were collected at sacrifice, weighed, and recorded. Small intestine samples were then homogenized in 1 mL of RIPA buffer with a protease inhibitor tablet (Complete Mini, EDTA-Free; Roche) and 1 mM EDTA and cooled on ice for 30 minutes. After 30 minutes, all samples were centrifuged at 10,600*g* for 10 minutes at 4°C and the resulting supernatant was collected. Sample supernatant total protein concentrations were determined using the Pierce 660 nm protein assay (Thermo Scientific). All homogenates were standardized to use 40 μg of protein. All ELISA assays were performed with commercial immunoglobulin ELISA assay kits following manufacturer guidelines with some modifications (Ready-SET Go! Kit, mouse IgA, IgG, IgE, IgM; eBioscience). Serum IgA, IgE, and IgM dilutions were performed at 1:100,000. Serum IgG dilution was performed at 1:50,000. Gut-homogenate dilution was performed at 1:5000. All samples were performed in duplicate.

### Intraepithelial lymphocyte isolation and processing.

At the time of sacrifice, the small intestine, from the proximal ileum to the distal jejunum, was harvested and freed from the mesentery. Peyer’s patches were removed from each sample before processing. The intestine was cut into several 1.5 cm pieces and added to a 50 mL conical tube containing 30 mL HBSS (Mediatech) with 5% FBS and 120 μL EDTA (Affymetrix). The sample was shaken for 20 minutes at 250 rpm at 37°C. This process was repeated. The intestinal samples were cut into 30 to 40 pieces and then added to 20 mL HBSS with 10% FBS, collagenase IV (1.38 mg/mL), and 8 μL of DNase. The sample was shaken for 11 minutes at 200 rpm at 37°C. The solution was strained, resuspended in HBSS with 5% FBS, and poured over glass wool slowly to remove debris. A Percoll gradient was used to further isolate lymphocytes. The resulting sample was then plated and stained according to the aforementioned protocols.

### Phagocytosis assay.

Phagocytosis assay with FITC-conjugated K-12 *E*. *coli* particles was performed per manufacturer’s specifications (Vybrant phagocytosis assay kit; ThermoFisher) with slight modifications. Briefly, whole blood was collected in heparinized syringes and 100 μL of blood was added to 10 μL of heparin in a flow cytometry tube. To the blood was added 50 μL of FITC-conjugated K-12 *E*. *coli* particles, and the samples were incubated at 37°C for 2 hours, protected from light. At 90 minutes into the incubation, anti-CD3, anti-GR-1, and anti-CD11c Abs were added to each sample 1:100. After 2 hours, RBCs were lysed and the samples were washed, resuspended, and analyzed by flow cytometry.

To measure nonspecific phagocytic capacity, a phagocytosis assay with phycoerythrin-conjugated (PE-conjugated) beads was performed per manufacturer’s specifications (Phagocytosis Assay Kit [IgG PE]; Cayman Chemical). Whole blood was collected into heparinized syringes from cardiac puncture and 100 μL of blood was added to 100 μL of 37°C RPMI 10% FBS in a flow cytometry tube. PE-conjugated beads (2 μL) were added and the samples were incubated at 37°C for 2 hours, protected from light. After incubation, RBCs were lysed and the samples were washed and resuspended. Samples were then stained with anti–GR-1 at a concentration of 1:100. Samples were washed and then resuspended in 200 μL of trypan blue–containing assay buffer and then analyzed via flow cytometry.

### Neutrophil total oxidative burst.

Whole blood was collected into heparinized syringes from cardiac puncture and 50 μL of blood was added to 550 μL of 37°C HBSS in Eppendorf centrifuge tubes. To each tube, 21 μL (5 mM) Rhodamine-123 (Thermo Scientific) and 6 μL of 100 nM W-peptide (WKYMVm–NH2; Anaspec) were added and incubated for 20 minutes at 37°C. Samples were then placed in an ice bath for 10 minutes. RBCs were then lysed and the samples were washed and resuspended. Samples were stained with anti–GR-1 at a concentration of 1:100. Samples were washed and then resuspended in FACS solution and analyzed via flow cytometry.

### Mitochondrial oxidative burst.

Whole blood was collected into heparinized syringes from cardiac puncture and 50 μL of blood was added to 550 μL of 37°C, 5 μM MitoSOX Red mitochondrial superoxide indicator (Thermo Scientific) solution. Samples were then incubated at 37°C for 20 minutes. Samples were then placed in an ice bath for 10 minutes. RBCs were then lysed and the samples were washed and resuspended. Samples were then stained with anti–GR-1 and anti-TLR4 at a concentration of 1:100. Samples were washed and then resuspended in FACS solution and analyzed via flow cytometry.

### Neutrophil depletion.

WT and JAM-A^–/–^ mice received an i.p. injection of anti-Ly6G Ab (250 μL of 1 mg/mL, clone 1A8; BioXcell) 24 hours before CLP.

### Statistics.

Statistical analysis software (GraphPad Prism 7.0) was used to analyze results, and values are presented as mean ± SEM. Each data set was first tested for Gaussian distribution of samples. If data were normally distributed, comparisons were performed with a 1-tailed *t* test. If data did not meet normality definitions, comparisons were done with a Mann-Whitney test. The ANOSIM test, within the *Vegan* package for R, was used to measure β diversity of the microbiome. For survival study comparisons, data were analyzed by log-rank test. *P* < 0.05 was predetermined as defining statistical significance.

### Study approval.

All studies were performed in accordance with the NIH Guidelines for the Use of Laboratory Animals and were approved by the IACUC at Emory University School of Medicine (Protocol 201800033).

## Author contributions

NJK and CMC conceived of and designed the study and drafted the manuscript. NJK, KTF, DAS, JMRB, JDL, MM, KMR, ZL, and EMB acquired the data. NJK, KTF, DAS, JMRB, JDL, CC, EMB, CAP, MLF, CMC analyzed and/or interpreted the data. KTF, DAS, JMRB, JDL, CC, MM, KMR, ZL, EMB, CAP, and MLF critically revised the manuscript.

## Supplementary Material

Supplemental data

## Version 1 07/12/2022

In-Press Preview

## Version 2 08/22/2022

Electronic publication
