# Supplementary material for: Junctional adhesion molecule-A deletion increases phagocytosis and improves survival in a murine model of sepsis
Source: JCI Insight. 2022 Aug 22;7(16):e156255. doi: 10.1172/jci.insight.156255 (PMC9462501; doi:10.1172/jci.insight.156255)
Supplement: Supplemental data [file jciinsight-7-156255-s030.pdf]

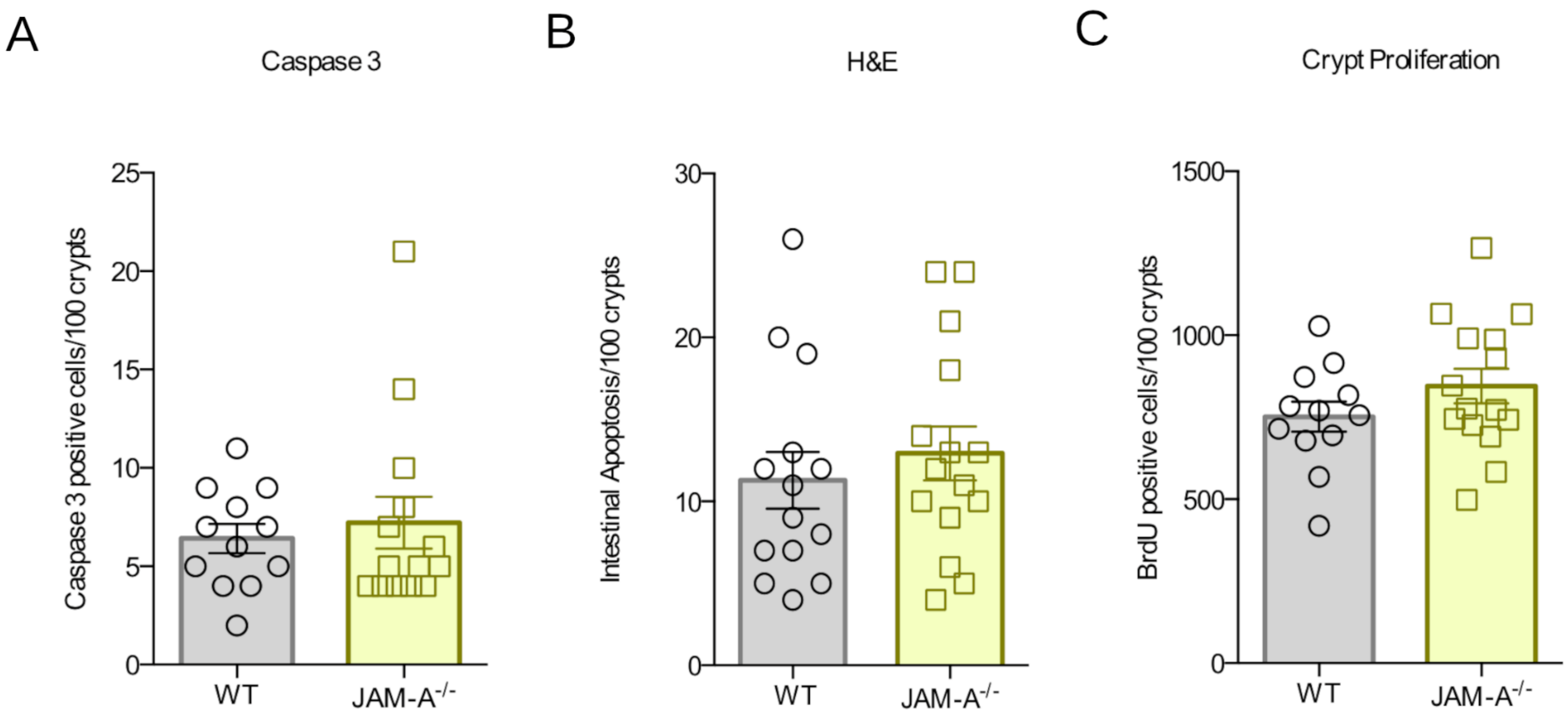

Supplemental Figure 1 (A, B) Apoptosis as measured by caspase 3 (p=0.77, n=12 WT, n=14 JAM-A$^{-/-}$) and H&E (p=0.49, n=14 WT, n=15 JAM-A$^{-/-}$) and **(C)** proliferation (p= 0.20, n=12 WT, n=15 JAM-A$^{-/-}$) were similar between septic JAM-A$^{-/-}$ and WT mice. T-test and Mann Whitney test for A-C depending on presence of Gaussian distribution.

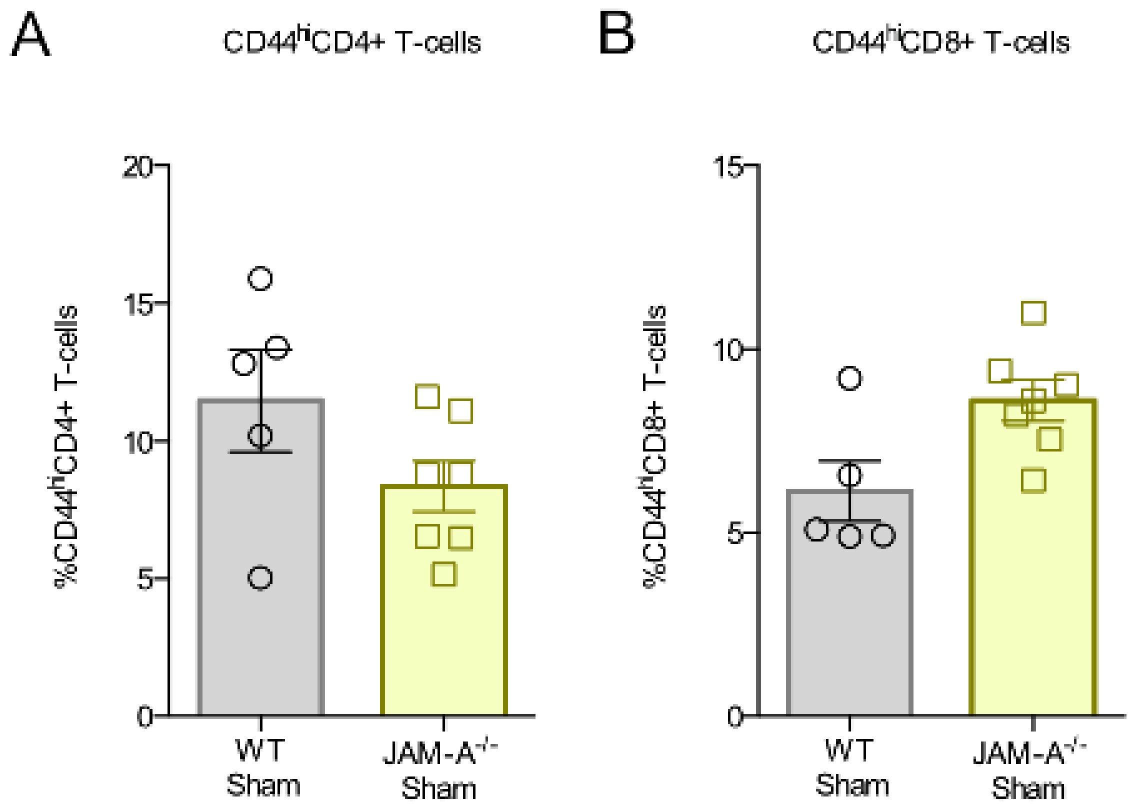

Supplemental Figure 2 (A,B) Percentages of both memory CD44^{hi}CD4^{+} T cells (p=0.20, n=5 WT, n=7 JAM-A$^{-/-}$) and memory CD44^{hi}CD8^{+} T cells (p=0.07, n=5 WT, n=7 JAM-A$^{-/-}$) were similar between septic JAM-A$^{-/-}$ and WT mice. Mann Whitney test for A, B.

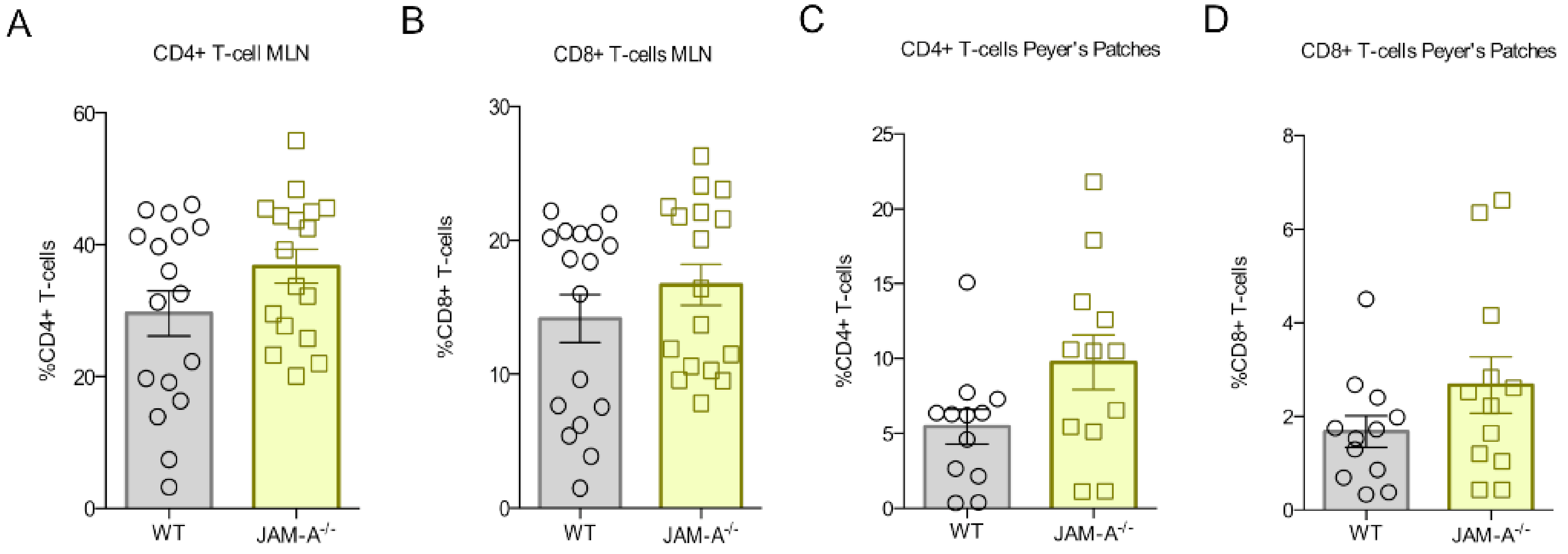

Supplemental Figure 3 (A-D) Percentages of bulk $CD4^+$ T cells and $CD8^+$ T cells were similar between septic JAM-$A^{-/-}$ and WT mice in both MLNs (p=0.11 and p =0.16 respectively, n=17 WT, n=17 JAM-$A^{-/-}$) and Peyer's Patches (p=0.06 and p=0.24 respectively, n=12 WT, n=12 JAM-$A^{-/-}$). T-test and Mann Whitney test for A-D depending on presence of Gaussian distribution.

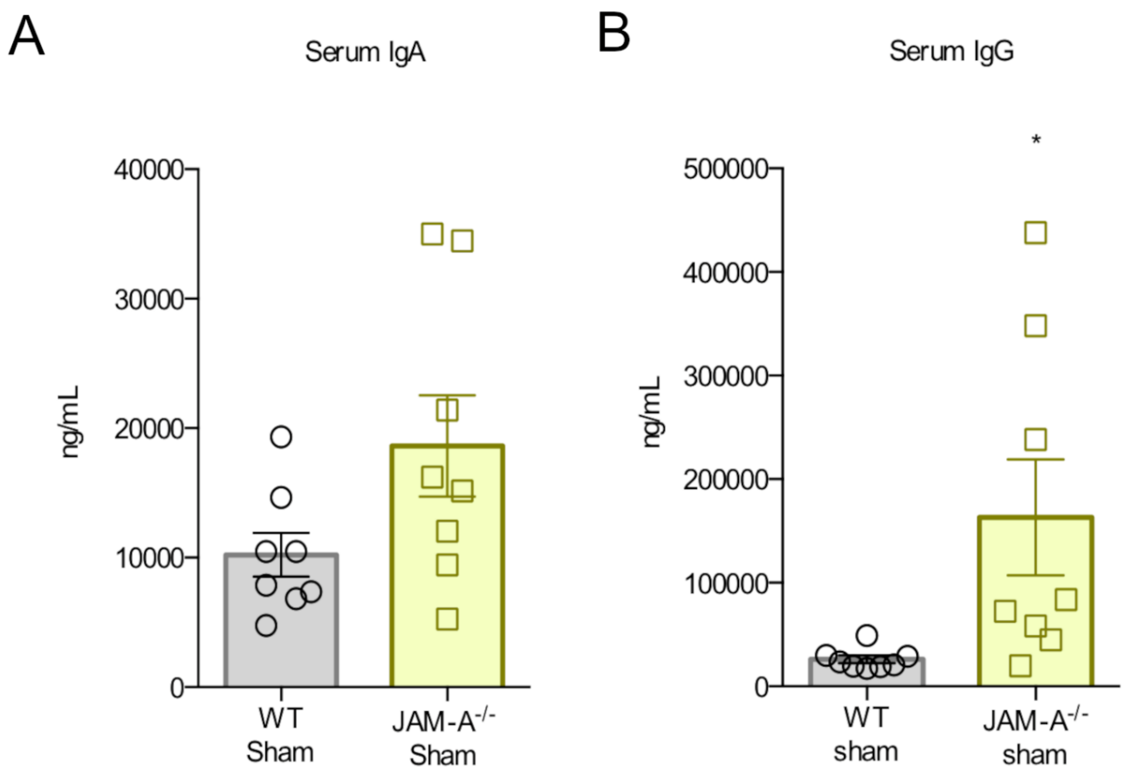

Supplemental Figure 4 (A) No statistically significant difference was found in serum IgA levels between sham JAM-A$^{-/-}$ and WT mice (p=0.07, n=8 WT, n=8 JAM-A$^{-/-}$). **(B)** In contrast, serum IgG levels were higher in sham JAM-A$^{-/-}$ mice (p=0.005, n=8 WT, n=8 JAM-A$^{-/-}$). T-test and Mann Whitney test for A, B depending on presence of Gaussian distribution.

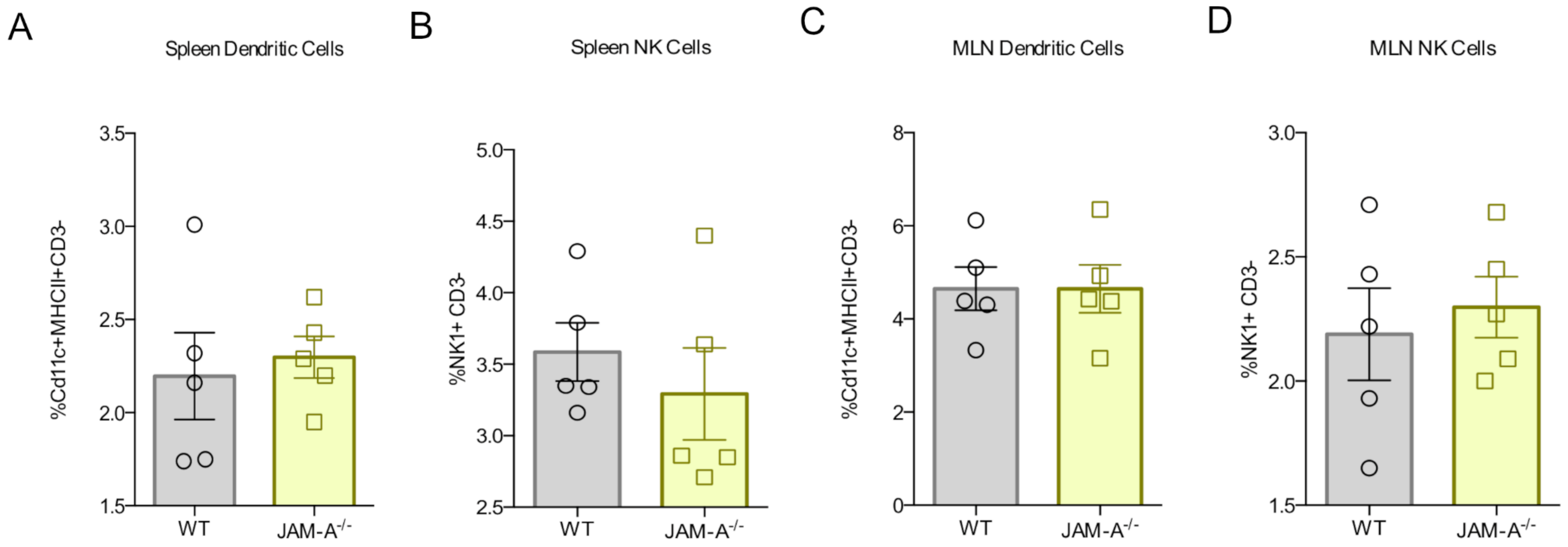

Supplemental Figure 5 (A-D) Percentages of dendritic cells and NK cells were similar between septic JAM-A$^{-/-}$ and WT mice in both spleen (p =0.53 and p=0.41, n=5 WT, n=5 JAM-A$^{-/-}$) and MLNs (p=0.94, and p=0.67, n=5 WT, n=5 JAM-A$^{-/-}$). Mann Whitney test for A-D.

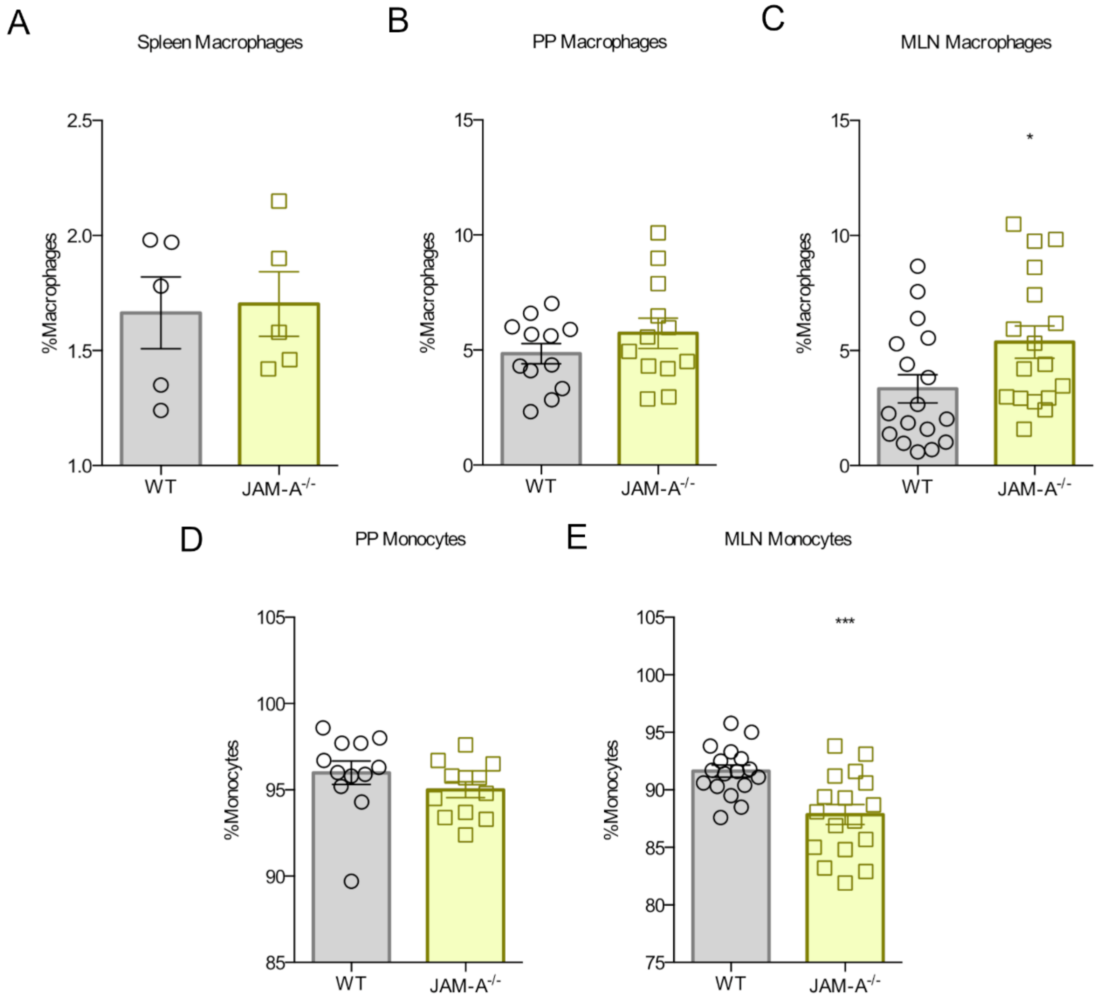

Supplemental Figure 6 (A-C) Percentages of macrophages were similar between septic JAM-A$^{-/-}$ and WT mice in spleen (p=0.84, n=5 WT, n=5 JAM-A$^{-/-}$) and Peyer's Patches (p=0.27, n=12 WT, n=12 JAM-A$^{-/-}$). In contrast, percentage of macrophages were higher in MLNs of JAM-A$^{-/-}$ mice (p =0.02, n=17 WT, n=17 JAM-A$^{-/-}$). **(D, E)** Percentages of monocytes were similar between septic JAM-A$^{-/-}$ and WT mice in Peyer's Patches (p=0.07, n=12 WT, n=12 JAM-A$^{-/-}$) whereas they were lower in knockout mice in MLNs (p=0.002, n=17 WT, n=17 JAM-A$^{-/-}$). T-test and Mann Whitney test for A-E depending on presence of Gaussian distribution.

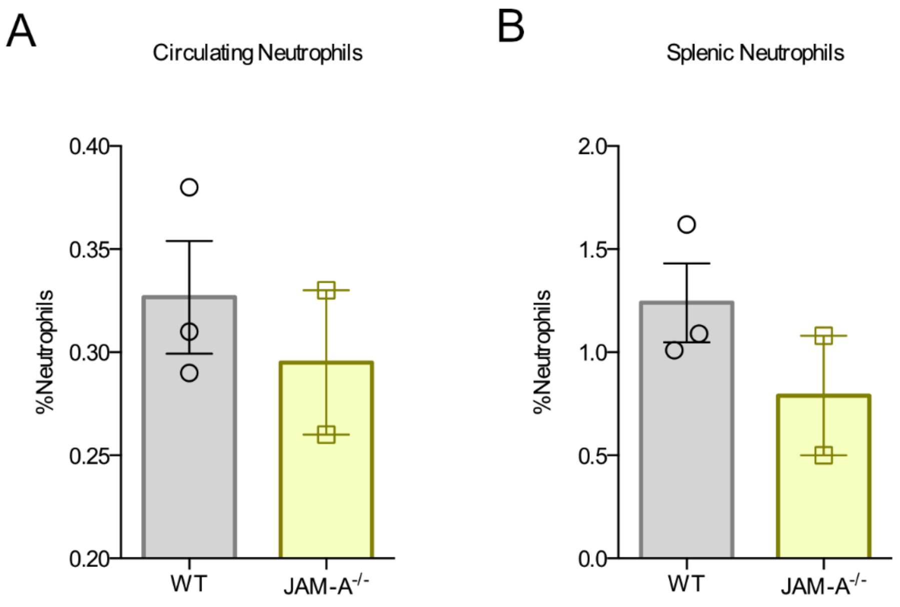

Supplemental Figure 7 (A, B) Ly6G depleting antibody given to WT and JAM-$A^{-/-}$ mice 24 hours prior to sepsis effectively depletes neutrophils in both the blood and spleen (n=3 WT, n=3 JAM-$A^{-/-}$).

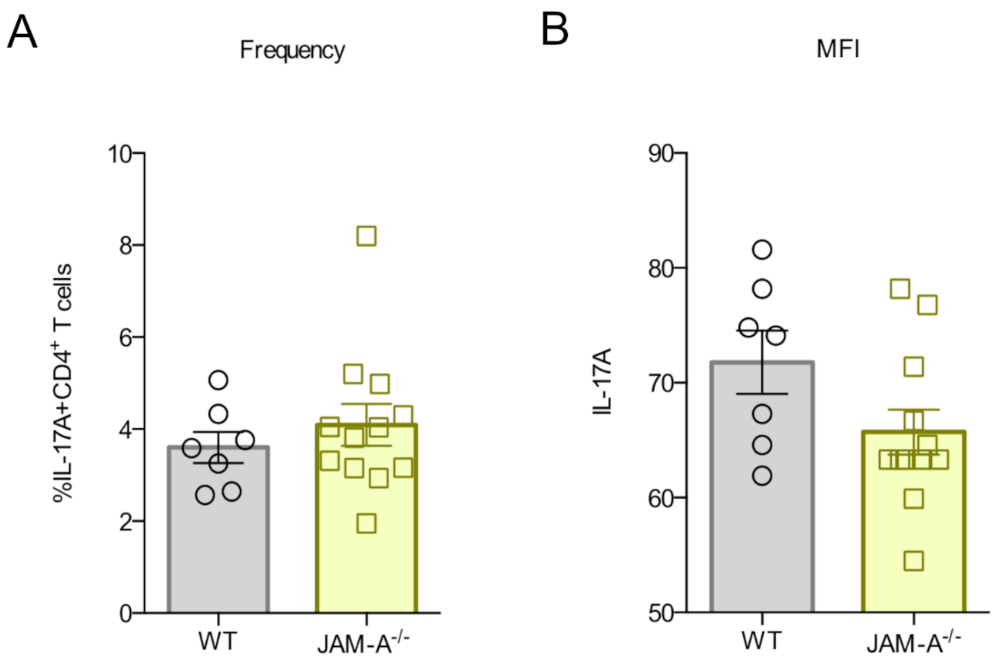

Supplemental Figure 8 (A-B) Frequency of splenic IL-17 was similar between septic JAM-A$^{-/-}$ and WT mice in bulk CD4^{+} cells (Frequency, p =0.59, mean fluorescence intensity, p=0.09, n=7 WT, n=12 JAM-A$^{-/-}$). Mann Whitney test for A-B depending on presence of Gaussian distribution.
